# Perspective-taking in video advertising: insights from EEG connectivity and channel-wise spectral analysis

**DOI:** 10.3389/fnbeh.2026.1740368

**Published:** 2026-07-24

**Authors:** Marco Bilucaglia, Chiara Casiraghi, Vincenzo Russo, Margherita Zito

**Affiliations:** 1 Department of Business, Law, Economics and Consumer Behaviour “Carlo A. Ricciardi,” Università IULM, Milan, Italy; 2 Behavior and Brain Lab IULM—Neuromarketing Research Centre, Università IULM, Milan, Italy

**Keywords:** advertising effectiveness, consumer neuroscience, electroencephalography (EEG), food and beverage communication, graph-theoretical analysis, neuromarketing, perspective-taking, social cognition

## Abstract

Perspective-taking, a key social cognition process, plays a significant role in shaping consumer behavior. Despite its relevance, existing research has largely focused on its visuo-spatial dimensions, with little attention given to its cognitive and emotional aspects, especially in the context of communication content perception, where no established electroencephalography (EEG) measures have assessed these components. In this exploratory study, EEG data were recorded from participants while viewing food and beverage advertisements (ads) featuring product interactions. They also provided ratings of their emotional and cognitive perspective-taking during the viewing, as well as their assessments of ad effectiveness. Channel-wise spectral powers and connectivity measures from both original and Minimum Spanning Tree networks were computed and correlated with self-report ratings. Results showed that cognitive perspective-taking correlated negatively with frontal θ power and positively with α clustering coefficient. Perceived ad effectiveness positively correlated with α clustering coefficient, path length, and degree divergence, as well as with δ path length and average degree. Although exploratory, this study provides initial evidence of EEG correlates of consumers' perspective-taking processes and suggests potential associations with advertising effectiveness. These findings may inform future hypotheses on cognitive load, working memory, and reward-related processes, which should be directly tested in confirmatory studies.

## 1 Introduction

Social cognition encompasses all those mental processes that enable individuals to understand, interpret, and respond to others' thoughts, intentions, and behaviors. These abilities are essential in complex social contexts and play a central role in influencing decision-making in our daily social interactions (Frith, 2008). Indeed, from an evolutionary perspective, they have developed to guide humans through the often unpredictable emotional, cognitive, and behavioral motives of others, thus promoting effective collaboration and social cohesion (Freeberg et al., 2019).

Within the wide spectrum of social cognition processes, *perspective-taking* refers to the ability to understand others by adopting their individual perspective and point of view (Quesque et al., 2024). This process comprises multiple components: *cognitive* perspective-taking involves reasoning about others' thoughts, beliefs, and intentions, while *emotional* perspective-taking relates to understanding their emotions and feelings (Healey and Grossman, 2018). In addition, *visuo-spatial* perspective-taking concerns the perceptual ability to imagine how the world appears from another person's visual point of view (Michelon and Zacks, 2006).

Perspective-taking not only influences social interactions, driving individuals to mimic traits and behaviors of a target person (Erle and Topolinski, 2017), but can also shape how we engage with media and communication content. For example, in the context of video advertisements (ads), adopting a character's point of view and understanding his/her emotions and cognitions may enhance overall ad liking (Chan et al., 2023). Indeed, research shows that when ads portray characters physically interacting with products—such as eating or drinking—viewers tend to mentally represent these actions by adoptingthe character's perspective (De Graaf et al., 2011), a simulation that could enhance message acceptance (Wojcieszak and Kim, 2016) and increase subjective craving for the advertised product (Palcu et al., 2019). This suggests that perspective-taking may act as a mechanism underlying advertising effectiveness, promoting both engagement with the ad, its message, and a more positive product perception. This view is also supported by theoretical models such as the Narrative Transportation Theory, which emphasizes the role of character identification and immersion in shaping persuasive communication (Green and Brock, 2000). Within this framework, perspective-taking can be seen as a mean for narrative transportation, deepening identification, involvement, and finally increasing message sensitivity and acceptance. In this sense, perspective-taking can be regarded as one of the psychological processes to be examined within Consumer Neuroscience, a field that aims to explore the neural and physiological mechanisms underlying consumer perception, emotion, and decision-making processes (Plassmann et al., 2015). Adopting a consumer neuroscience approach to study perspective-taking may, therefore, help develop integrative models of consumer response to advertising, as well as other communication and marketing content, linking cognitive and affective processes to behavioral outcomes.

Due to its high relevance across different contexts, neuroscientific research has explored the neural basis of perspective-taking processes, particularly through functional Magnetic Resonance Imaging (fMRI), and has identified a distributed network of brain regions involved in perspective-taking (Van Der Heiden et al., 2013; Healey and Grossman, 2018; Jääskeläinen et al., 2021). These findings provide a well-established neural framework for understanding perspective-taking processes, which can also inform studies with other neuroimaging techniques, such as electroencephalography (EEG). Within consumer neuroscience, EEG is indeed a popular method for its high temporal resolution, non-invasiveness, and portability that make it well-suited for investigating consumers' real-time cognitive and emotional responses to several types of stimuli, such as advertisements (Alsharif and Isa, 2024).

Spectral analysis is one of the most established EEG analysis approaches. It involves computing spectral measures within conventional bands, most commonly delta (δ), theta (θ), alpha (α), beta (β), and gamma (γ) (Matthis et al., 1981).

When applied to specific scalp regions or electrode sites, these measures can reflect distinct cognitive and emotional processes, such as, among others, short-term (Onton et al., 2005) and long-term memory (Babiloni et al., 2004), mental fatigue (Tran et al., 2020), motivational direction (Davidson et al., 1990), sustained attention (Ko et al., 2017), and cognitive engagement (Kennedy and Hassin, 2019). Assessing these processes through EEG spectral analysis can also bring significant insights to consumer research. For example, in the specific context of ad viewing and perception, a decrease of parietal α power has been linked to increased attentional focus (Shestyuk et al., 2019; Kolar et al., 2021), and frontal α power asymmetry to an individual's tendency to approach or withdraw from the ad (Ohme et al., 2010; Shestyuk et al., 2019). Power in the θ band has been associated with ad memorization and recall (Vecchiato et al., 2014), and the ratio between β power and the sum of α and θ power across all EEG channels has been used as a measure of cognitive engagement with the ad (Russo et al., 2023). EEG spectral analysis has also been adopted to assess the impact of specific ad features, such as the narrative structure, on consumer responses (Wang et al., 2016). These EEG-based metrics have proven to be highly effective in assessing consumers' responses to media content, even predicting outcomes in out-of-sample populations (Boksem and Smidts, 2014; Venkatraman et al., 2014).

Nonetheless, comprehensively assessing consumer cognition, especially when it involves more complex functions such as perspective-taking, may require more complex analyses. Approaches based on connectivity, for instance, can provide a broader, richer, network-level view that complements traditional frequency-based EEG measures (Chiarion et al., 2023).

Connectivity analysis aims to capture the statistical dependencies between brain signals ${\left\{x_{i}\left(t\right)\right\}}_{i=1}^{N}$ across time, frequency, or information domains (Chiarion et al., 2023). This is typically achieved by means of pairwise connectivity measures *c*_*ij*_:*x*_*i*_(*t*) × *x*_*j*_(*t*) → ℝ, which are organized into a square adjacency matrix $X={\left\{c_{ij}\right\}}_{i,j=1}^{N}$. This matrix represents a brain network, where *c*_*ij*_ is represents the edge connecting nodes *i* and *j* (Hassan and Wendling, 2018). The Minimum Spanning Tree (MST) provides a simplified yet informative representation of the brain network. It is constructed by selecting the minimal set of edges required to preserve the network's overall connectivity without forming cycles (Centeno et al., 2022). While preserving essential features such as edge weights and network density, the MST enhances comparability across individuals and studies (Tewarie et al., 2015). To describe the properties of both the original and MST-based brain network representations, several topological measures have been proposed. The formers include average degree *A* (the mean connectivity), clustering coefficient *C* (a measure of functional segregation), and characteristic path length *L* (a measure of functional integration) (Rubinov and Sporns, 2010). For MST-based networks, key measures include leaf fraction *LF* (percentage of end nodes), diameter *D* (largest distance between two nodes), three hierarchy *TH* (the balance between large-scale integration and overload of central nodes), and degree divergence *K* (reflecting the broadness of the edge distribution) (Fraschini et al., 2016; Tewarie et al., 2015).

Brain connectivity measures have been widely applied in both clinical and experimental research settings (Cao et al., 2021), and have shown great potential in various research fields, from clinical populations (Blomsma et al., 2022) to memory processes (Nuamah and Uba, 2024), mental fatigue (Chen et al., 2018), and emotion recognition (Liu et al., 2022; Yin et al., 2022). However, although this framework has recently been applied within the fields of consumer neuroscience and advertising research (Adalarasu et al., 2025), it has not yet been employed in this context to investigate social cognitive processes such as perspective-taking. While traditional EEG metrics target specific and localized oscillatory brain patterns, connectivity analyses capture the activity of distributed networks, offering a more suitable approach for studying complex phenomena such as perspective-taking. Thus, applying this approach to advertising research could provide new insights into the neural basis of consumers' social perception and its relationship with ad effectiveness.

The limited EEG research on perspective-taking has indeed largely relied on traditional approaches, such as spectral analysis and event-related potentials, and has mainly focused on visuo-spatial aspects (Rochas et al., 2023, 2024; Gunia et al., 2024) or on specific populations, such as young children (Meyer et al., 2024). The findings cannot, thus, be generalized to adult or consumer populations, nor to the cognitive and emotional dimensions of perspective-taking, particularly in the context of advertising perception and evaluation. This gap highlights the need to expand EEG research to include connectivity-based methodologies, particularly to more accurately assess the role of social cognition processes in consumer behavior.

This exploratory study aims to connect the gaps from social cognitive neuroscience and consumer research by addressing the following research objectives:

1. Investigate how cognitive and affective measures of perspective-taking elicited by testimonials in video advertisements correlate to EEG-based spectral and connectivity measures.
2. Investigate how measures of perceived advertisement effectiveness correlate to EEG-based spectral and connectivity measures.
3. Investigate whether EEG-based spectral and connectivity measures of perceived advertisement effectiveness overlap with those related to cognitive and affective perspective-taking, potentially suggesting the recruitment of shared rather than distinct neural processes.

Given the exploratory nature of the study and the lack of prior research employing EEG connectivity measures in this specific context, precise a priori hypotheses could not be developed. To address the research objectives, EEG data were collected while participants viewed testimonial-style video advertisements featuring product interactions. From these recordings, channel-wise spectral powers and functional connectivity measures (from both raw and MST networks) were computed and correlated with self-report measures of perspective-taking and ad effectiveness, to finally explore potential associations between brain activity and consumer social cognition processes.

## 2 Methods

The study was approved by the IULM University Ethics Committee (prot. n. 0067815) and complied with both the Declaration of Helsinki and the European General Data Protection Regulation (GDPR).

### 2.1 Sample

Twenty participants (10 females, 10 males), aging between 27 and 55 years (*M* = 41.32, *SD* = 8.80) took part in the study. This sample size is consistent with those typically employed in exploratory consumer neuroscience research (Garczarek-Bak et al., 2021). A sensitivity analysis performed using G*Power (Faul et al., 2007), considering one group, four measures and standard parameters (α = 0.10, 1−β = 0.80, ρ = 0.5, *andϵ* = 1) gave a minimum detectable effect size of *f* = 0.270, interpreted as “small-to-medium” (Cohen, 1992). This was in line with the median effect size in both psychology and consumer neuroscience literature, which ranges from “small-to-medium” to “more-than-large” (Szucs and Ioannidis, 2017; Schäfer and Schwarz, 2019). The gender distribution of the sample was balanced in terms of mean age, with no significant differences between females and males [*t*_(17)_ = 1.889, *p* = 0.076]. Participation was voluntary. Written informed consent was obtained from each participant before the start of the experiment.

### 2.2 Stimuli

The stimuli consisted of 30 s ads (standard television advertising format), selected from YouTube and released within the last 5 years. To avoid comprehension difficulties and control for language knowledge variables, they did not include any spoken dialogue, with the only audio being the ad's background music. The ads advertised food (chocolate—AD_1_, chips—AD_3_) and beverage (beer—AD_2_, gin—AD_4_) products. They featured testimonial-style interactions with the products, with a female testimonial in AD_1_ and AD_4_ and a male testimonial in AD_2_ and AD_3_. In the food-related ads (AD_1_, AD_3_), the testimonials were shown eating the product, while in the beverage-related ads (AD_2_, AD_4_), they were seen drinking it.

Rather than focusing on the entire ads, only the specific interaction segments were considered, as they were expected to elicit stronger perspective-taking responses than those showing the product alone. The interaction segments varied in length, with an average duration of 3.11 ± 1.18*s* (range: 1.34–4.9s). For brevity, the segments will be hereafter referred as AD_1 − 4_.

### 2.3 EEG instrumentation

The EEG was recorded via NVX-36 device (Medical Computer Systems, Ltd., Moscow, Russia) from 22 Ag/AgCl scalp electrodes placed according to the 10–10 system (Nuwer, 2018), 2 Ag/AgCl ear clips (A1, A2), and 1 Ag/AgCl adhesive patch positioned on the left mastoid (M1). The montage was monopolar, internally referenced and grounded to M1. Data were collected through NeoRec software (Medical Computer Systems, Ltd.) at a sample frequency of 2*KHz* and a vertical resolution of 24 bits. To keep electrode impedance below the recommended threshold of 20*kΩ* (Sinha et al., 2016), the skin was initially scrubbed with the NuPrep cream (Spes Medica, Srl, Genoa, Italy). Then, the Neurgel conductive gel (Spes Medica, Srl) was applied.

iMotions software (iMotions A/S) was used to deliver the stimuli, the instructions, and to collect the survey responses. Data synchronization was ensured by a TTL pulse generated by iMotions at the study beginning and fed into the NVX-36 through the ESB - EEG synchronization box (Bilucaglia et al., 2020).

Data processing and statistical analyses were carried out in Matlab (The Mathworks, Inc., Natick, MA, USA), using EEGLab (Delorme and Makeig, 2004) and Brain Connectivity Toolbox (Rubinov and Sporns, 2010).

### 2.4 Self-report variables

Perceived advertisement effectiveness (EFF), emotional perspective-taking (EMO), and cognitive perspective-taking (COG) were assessed through self-report scales (Palcu et al., 2019). EFF captures the ability of the advertisement to effectively promote the product. EMO reflects the extent to which participants experience the emotions expressed by the testimonials, while COG refers to the extent to which they experience the cognitive and sensory experience portrayed. EFF consisted of five items assessing ad effectiveness and consumer-related behaviors (e.g., “*The advertisement is effective in promoting the product*”). EMO and COG were measured with ten items assessing participants' emotional involvement with the testimonials (EMO; e.g., “*It seemed I could feel the actors' emotions as they ate/drank*”) and their cognitive and sensory perceptions (COG; e.g., “*It seemed I could feel the texture of the food/drink*”).

All the items were rated on a Likert scale from 1 (“not at all”) to 6 (“very much”), omitting the middle option to discourage neutral responses (Taherdoost, 2019). Moreover, to avoid forced responses reducing data reliability (Sischka et al., 2020), participants were not required to respond to the items if they were unsure of their evaluation. Missing responses were then handled via pairwise deletion.

### 2.5 Experimental procedure

**Figure 1:**
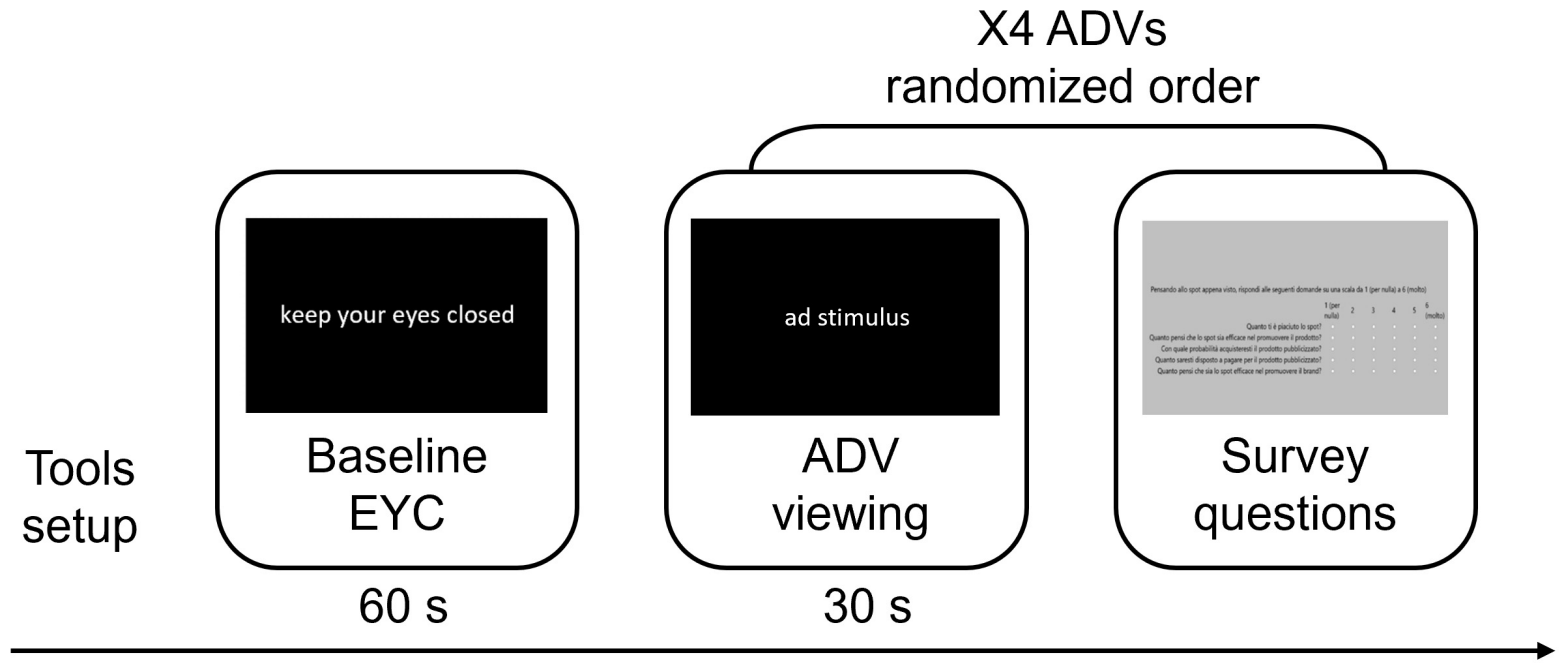
Overview of the study's experimental protocol.

After the EEG setup—see Fici et al. (2024) for additional information on the procedure—participants were seated in front of a 23.8^′′^ display monitor at a distance of approximately 60–70 cm, and were provided with a mouse to respond to the self-report questions. Ambient light and temperature were kept constant across participants. The experimental protocol (Figure 1) began with a 60*s* baseline period, during which participants were instructed to keep their eyes closed (EYC). After this, participants viewed all 4 ad stimuli, presented in a randomized order. After each ad, participants completed self-report scales to assess EMO, COG, and EFF dimensions. The entire experimental procedure lasted approximately 20 mins per participant.

### 2.6 Data pre-processing

The EEG was re-referenced to the linked A1–A2, resampled to 512 Hz and band-pass filtered (0.1–40 Hz IV order zero-phased Butterworth's filter). Sinusoidal power line noise (50 and 100 Hz) was removed by means of CleanLine's multi-taper-based regression method (Bokil et al., 2010) and non-stationary artifacts were corrected through the Artifact's Subspace Reconstruction (Chang et al., 2020) with standard cutoff parameter (κ = 20). Independent Component Analysis based on the SOBI algorithm (Urigüen and Garcia-Zapirain, 2015) was performed on a heavily filtered (1–30 Hz IV order zero-phase Butterworth's filter) and resampled (100 Hz) surrogate of the data. The computed weight matrix was multiplied by the original data to obtain the Independent Components (ICs), as in Bilucaglia et al. (2024). Artifactual ICs were identified by means of the ICLabel classifier (Pion-Tonachini et al., 2019) as those with probability of “not-brain” P≥0.9 and removed. Non-artifactual ICs were then back-projected to the sensor space. Finally, the Current Source Density transformation was performed to improve the spatial resolution (Kayser and Tenke, 2015). The cleaned EEG was offline aligned to the starting TTL pulse and epoched according to the experimental phases (i.e., EYC baseline and interaction segments AD_1 − 4_).

As an index of signal quality, we tracked the number of artifactual ICs identified during data pre-processing. Among 19 of the 20 participants, the mean number of artifactual ICs was 2.37 ± 0.96 (range: 1–5). One participant, which showed more than 20 artifactual ICs, was excluded from subsequent analyses.

For each subject, the Individual Alpha Frequency (IAF) was computed as the center of gravity of the Power Spectral Density (PSD) in the extended (7.5–12.5 Hz) alpha band (Klimesch, 1999). The PSD consisted of the average “occipital PSD” and was computed in the EYC epoch following Welch's approach, with 1*s*−long Hamming windows and 50% of overlapping (Bilucaglia et al., 2022). The IAF allowed the definition of subject-specific δ, θ, α, β and γ bands, as (Borghini et al., 2019):

- δ = [0, *IAF*−6]
- θ = [*IAF*−6, *IAF*−2]
- α = [*IAF*−2, *IAF*+2]
- β = [*IAF*+2, *IAF*+16]
- γ = [*IAF*+16, *IAF*+25]

### 2.7 Self-report analysis

The EFF, COG, and EMO scales showed an overall internal reliability of α = 0.915 ± 0.053 (range: 0.802–0.975), exceeding the “good” threshold of α = 0.7 (Taber, 2017). The items were thus scale-wise averaged for subsequent correlation analyses. For brevity, the composite scores of each scale will be hereafter referred as COG, EMO and EFF. Table 1 shows the descriptive statistics of the scales, split per stimulus.

**Table 1:** Descriptive statistics of the scales, split per stimulus.

Stimulus	Scale	#	M	SD	Min	Max
AD_1_	COG	17	3.478	1.333	1.250	6.000
	EMO	17	3.412	1.564	1.000	6.000
	EFF	17	4.271	1.311	1.800	6.000
AD_2_	COG	17	3.706	1.498	1.000	5.750
	EMO	18	3.972	1.557	1.000	6.000
	EFF	18	4.267	1.131	1.800	5.800
AD_3_	COG	17	3.316	1.566	1.000	5.500
	EMO	17	3.529	1.473	1.000	6.000
	EFF	17	4.635	1.045	2.600	6.000
AD_4_	COG	18	2.431	1.421	1.000	5.250
	EMO	18	2.472	1.557	1.000	6.000
	EFF	18	3.067	1.501	1.000	5.600

COG, cognitive perspective-taking; EFF, perceived ad effectiveness; EMO, emotional perspective-taking; #, valid responses; M, mean; SD, standard deviation; Min, minimum; Max, maximum.

### 2.8 Spectral analysis

For each subject *i*, stimulus s = AD_s_, channel *c* and band *b* = {δ, θ, α, β, γ}, normalized spectral powers σ_*i, s, c, b*_ were computed as in the following Equation 1 (Bilucaglia et al., 2022):


$$\begin{array}{c} \sigma_{i,s,c,b}=\frac{\int_{b}P_{i,s,c}\left(f\right)df}{\int_{-\infty }^{+\infty }P_{i,s,c}\left(f\right)df} \end{array}$$ (1)

where *P*_*i, s, c*_(*f*) is the power spectral density of the signal *x*_*i, s, c*_(*t*) computed following the Welch's approach (1*s*−long Hamming windows and 50% of overlapping).

Spearman's correlations ρ_*s, c, b*_ between spectral powers and self-reports were performed as in the following Equation 2:


$$\begin{array}{c} \rho_{s,c,b}=\frac{\sum_{i=1}^{N}\left(\mathrm{rk}\left(\sigma_{i,s,c,b}\right)-{\bar{R}}_{\sigma_{s,c,b}}\right)\left(\mathrm{rk}\left(I_{i,s}\right)-{\bar{R}}_{I_{s}}\right)}{\sqrt{\sum_{i=1}^{N}{\left(\mathrm{rk}\left(\sigma_{i,s,c,b}\right)-{\bar{R}}_{\sigma_{s,c,b}}\right)}^{2}}\sqrt{\sum_{i=1}^{N}{\left(\mathrm{rk}\left(I_{i,s}\right)-{\bar{R}}_{I_{s}}\right)}^{2}}}, \end{array}$$ (2)

where *N* = 19 is the final sample size, σ_*i, s, c, b*_ is the power in band *b* from channel *c* of subject *i* corresponding to stimulus *s*, $I_{i,s}$ is the composite score (either EFF, EMO, or COG) of subject *i* corresponding to stimulus *s* and rk(·) is the rank operator. The mean ranks ${\bar{R}}_{\sigma_{s,c,b}}$ and ${\bar{R}}_{I_{s}}$ are given by the following Equation 3:


$$\begin{array}{c} \begin{array}{c} {\bar{R}}_{\sigma_{s,c,b}}=\frac{1}{N}\sum_{i=1}^{N}\mathrm{rk}\left(\sigma_{i,s,c,b}\right),\\ {\bar{R}}_{I_{s}}=\frac{1}{N}\sum_{i=1}^{N}\mathrm{rk}\left(I_{i,s}\right). \end{array} \end{array}$$ (3)

The obtained *p*-values were channel-wise corrected for multiple comparisons according to the FDR method (Benjamini and Hochberg, 1995) within each condition. The significance level was set at α = 0.10, consistent with the exploratory nature of the study and the small sample size (Schumm et al., 2013). This threshold was chosen to increase sensitivity to true effects, while acknowledging a higher false-positive rate. To quantify this trade-off, we conducted a post-hoc analysis in G*Power using the same repeated-measures design (one group, four measures, *N* = 20, ρ = 0.5, and ϵ = 1) and an effect size of *f* = 0.270. Under these assumptions, increasing α from the conventional 0.05 to 0.10 increased the nominal Type I error probability by five percentage points, while increasing sensitivity 1–β by eight percentage points (from 0.80 to 0.88).

### 2.9 Connectivity analysis

For each subject *i*, stimulus *s*, and band, *b* the adjacency matrix *X*_*i, s, b*_ = {*ciPL*_*V*_*i, s, b, j, k*_}*j, k*_ was computed, based on the following corrected-imaginary Phase Locking Values (ciPLV) (Bruña et al., 2018) given by the following Equation 4:


$$\begin{array}{c} ciPLV_{i,s,b,j,k}=\frac{\frac{1}{T}\sum_{t\in T}ℜ\left\{{\tilde{x}}_{i,s,b,j}\left(t\right){\tilde{x}}_{i,s,b,k}^{\prime }\left(t\right)\right\}}{\sqrt{1-{\left(\frac{1}{T}\sum_{t\in T}ℑ\left\{{\tilde{x}}_{i,s,b,j}\left(t\right){\tilde{x}}_{i,s,b,k}^{\prime }\left(t\right)\right\}\right)}^{2}}}, \end{array}$$ (4)

where ℜ{·} and ℑ{·} are the real and imaginary part, respectively, (·)′ is the complex-conjugate, *T* is the signal length and (*j, k*) = 1, …, 32 are the channel indices. The analytic signal ${\tilde{x}}_{i,s,b,j}\left(t\right)$ was obtained by first band-pass filtering (zero-phased IV order Butterworth's filter) the channel *k* in the band *b* and then applying the Hilbert-transform.

ciPLV was chosen due to its robustness against zero-lag effects associated with volume conduction or source leakages, which can dominate over real connectivities (Bruña and Pereda, 2021). Moreover, compared to similar robust phase-coupling measures, such as the Phase Lag Index (PLI), PLV also shows greater test-retest reliability (Garcés et al., 2016).

Once computed, the matrices *X*_*i, s, b*_ were first normalized to the [−1, 1] range and then thresholded by retaining the strongest 10% of edges, according to past studies (Josefsson et al., 2019; Youssef et al., 2021; Cai et al., 2023). Although potentially sub-optimal, this fixed, pre-specified approach represented a conservative choice, given the strong influence of threshold level on network measures and the lack of an established optimal criterion (Adamovich et al., 2022). Then, average degree *A*_*i, s, b*_, clustering coefficient *C*_*i, s, b*_ and characteristic path length *L*_*i, s, b*_ measures (Rubinov and Sporns, 2010) were calculated.

For each rescaled and thresholded matrix *X*_*i, s, b*_, the MST ${\hat{X}}_{i,s,b}$ was computed according to Kruskal's algorithm (Kruskal, 1956). Then, leaf fraction (*LF*_*i, s, b*_), diameter (*D*_*i, s, b*_), tree hierarchy (*TH*_*i, s, b*_) and kappa divergence (*K*_*i, s, b*_) (Fraschini et al., 2016) were calculated.

These measures are conventional descriptors in EEG-based connectivity analyses. *A*, *C*, and *L* capture complementary aspects of functional segregation and integration (Rubinov and Sporns, 2010), while the specific MST-based measures were computed as they avoid arbitrary thresholding and density bias, are insensitive to global strength yet sensitive to true topology changes, and are less affected by epoch length (Tewarie et al., 2015; Fraschini et al., 2016; Blomsma et al., 2022).

Similarly to the spectral powers, Spearman's correlations between the measures and the self-report items were computed and measure-wise corrected for multiple comparisons.

## 3 Results

For AD_1_ stimulus, COG showed a significant positive correlation with *C*_AD_1_, α_, that is, the clustering coefficient of the raw adjacency matrix in the α band *X*_AD_1_, α_ [*r*_(15)_ = 0.709, *p* = 0.050].

For AD_2_ stimulus, COG showed two significant negative correlations: with σ_AD_2_, *F*7, θ_, that is, the spectral power in the θ band at channel F7 [*r*_(15)_ = –0.708, *p* = 0.086], and with σ_AD_2_, *F*4, θ_, that is, the spectral power in the θ band at channel F4 [*r*_(15)_ = –0.705, *p* = 0.086].

For the AD_4_ stimulus, EFF showed five positive correlations. In the α band, it positively correlated with *C*_AD_4_, α_, that is, the clustering coefficient of the raw adjacency matrix *X*_AD_4_, α_ [*r*_(16)_ = 0.649, *p* = 0.036]; with *K*_AD_4_, α_, that is, the kappa divergence of the MST adjacency matrix [*r*_(16)_ = 0.641, *p* = 0.036); and with *L*_AD_4_, α_, that is, the characteristic path length of the MST adjacency matrix ${\hat{X}}_{{\text{AD}}_{4},\alpha }$ [*r*_(16)_ = 0.664, *p* = 0.036]. In the δ band, EFF resulted positively correlated with *A*_AD_4_, δ_, that is, the average degree of the raw adjacency matrix *X*_AD_4_, δ_ [*r*_(16)_ = 0.652, *p* = 0.036], and with *L*_AD_4_, δ_, that is, the characteristic path length of the MST adjacency matrix ${\hat{X}}_{{\text{AD}}_{4},\delta }$ [*r*_(16)_ = 0.595, *p* = 0.064].

No other significant correlations emerged between self-reports and EEG metrics, either at the channel or network level.

To further support the results, we performed a control analysis. For each stimulus AD_*i, s*_ (with *i* and *s* being, respectively, the ad and subject indices) we extracted a neutral pre-stimulus ${\bar{\text{AD}}}_{i,s}$, with the same length, drawn adjacently and preceding—still featuring the testimonial but not showing any interaction with the product. Matching duration controlled for the effects of window length in both network measures (Fraschini et al., 2016) and spectral estimates (van Vugt et al., 2007). Choosing an immediately preceding position served to preserve, as much as possible, the local audiovisual and narrative context, which has been shown to have an accumulative impact on the processing of naturalistic stimuli (Jääskeläinen et al., 2021). Then, spectral and network measures showing significant correlations in the target stimuli AD_*i, s*_ were extracted from the matched neutral windows ${\bar{\text{AD}}}_{i,s}$ and similarly correlated. No significant associations emerged (all *p* > 0.10, uncorrected).

**Figure 2:**
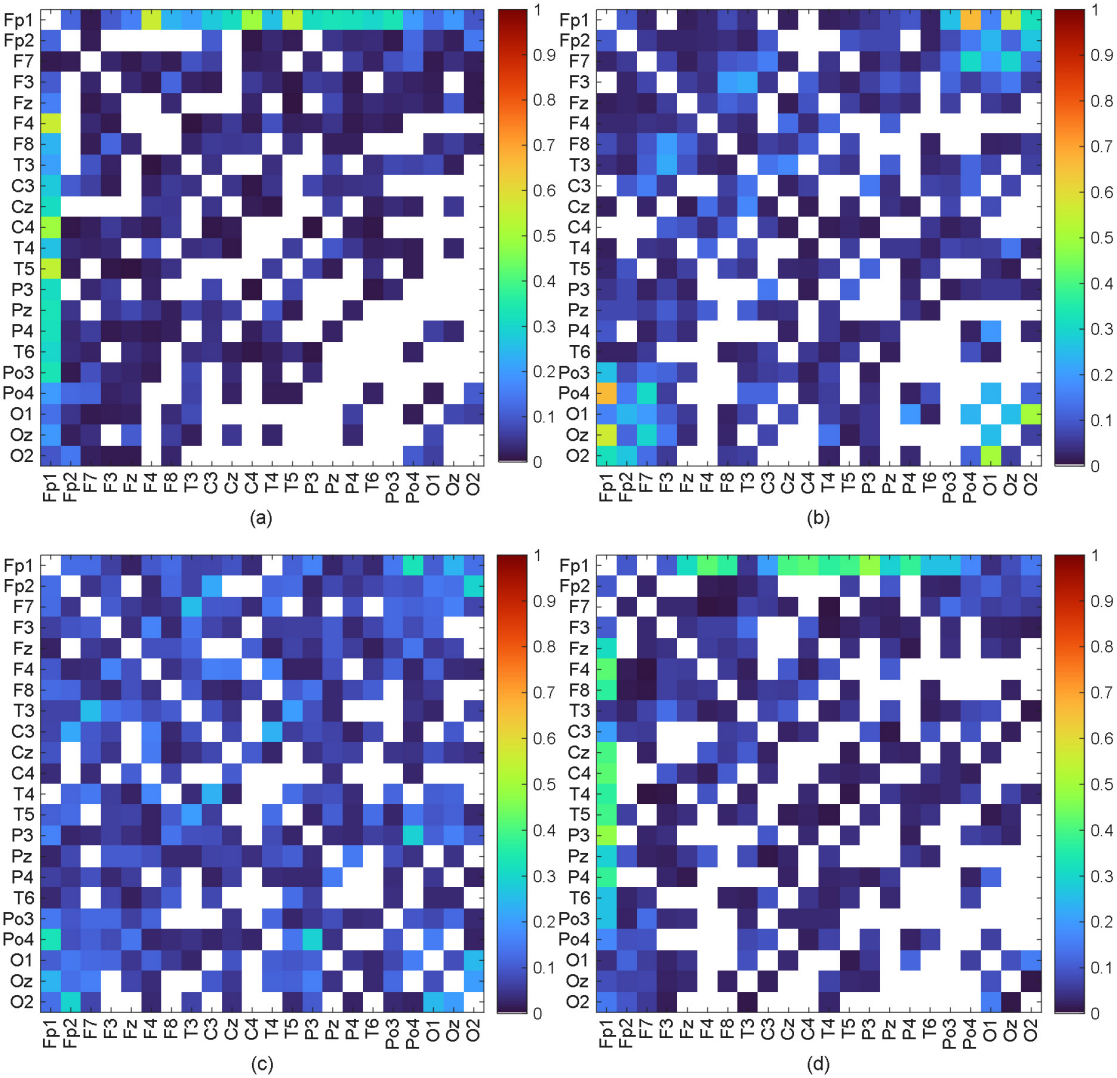
Rescaled and thresholded (top 10%) adjacency matrices averaged across subjects, with colors reflecting the strenghts of the edges: **(a)** raw adjacency matrix *X*_AD_1_, α_; **(b)** raw adjacency matrix *X*_AD_4_, α_; **(c)** MST adjacency matrix ${\hat{X}}_{{\text{AD}}_{4},\alpha }$; **(d)** raw adjacency matrix *X*_AD_4_, δ_.

Table 2 shows the correlations of stimuli (ρ) and matched neutral pre-stimuli $\left(\bar{\rho }\right)$, split per stimulus, scale, frequency band, and EEG measure. Figure 2 shows **(a)** the raw adjacency matrix *X*_AD_1_, α_, **(b)** the raw adjacency matrix *X*_AD_4_, α_, **(c)** the MST adjacency matrix ${\hat{X}}_{{\text{AD}}_{4},\alpha }$, and **(d)** the raw adjacency matrix *X*_AD_4_, δ_ – all averaged across subjects. Colors reflect the strength of the edges, subject-wise normalized in the [−1, 1] range and thresholded (top 10%). White represents a lack of connections.

**Table 2:** Spearman's correlations between self-report variables and EEG measures, for both interaction sequences (ρ) and neutral windows $\left(\bar{\rho }\right)$, including both channel-wise spectral power (σ, reported with electrode location) and connectivity measures (C, K).

Stimulus	Scale	Frequency band	EEG measure	ρ	$\bar{\rho }$
AD_1_	COG	α	C	0.709^*^	0.075
AD_2_	COG	θ	σ_*F*7_	−0.708^*^	0.185
			σ_*F*4_	−0.705^*^	−0.337
AD_4_	EFF	α	C	0.649^**^	0.225
			K	0.641^**^	0.424
			L	0.664^**^	0.415
		δ	A	0.652^**^	0.036
			L	0.595^*^	−0.041

Results are reported by frequency band, EEG measure, and stimulus.

COG, cognitive perspective-taking; EFF, perceived ad effectiveness; σ, channel-wise spectral power; C, clustering coefficient; K, kappa divergence. Significances are marked as ^*^p < 0.1,^**^p < 0.05.

## 4 Discussion

This exploratory study analyzed the relationship between cognitive and emotional perspective-taking, perceived ad effectiveness, and their corresponding EEG-based spectral and connectivity measures. EEG data were recorded while participants viewed food and beverage ads featuring testimonial-style product interactions (eating and drinking). Both channel-wise spectral powers and connectivity measures—computed from raw and MST adjacency matrices—were extracted and then correlated with participants' self-reports of cognitive and emotional perspective-taking and perceived ad effectiveness.

Regarding perspective-taking EEG correlates, the observed negative correlations between frontal θ powers (σ_*F*7, θ_ and σ_*F*4, θ_) and cognitive perspective-taking (COG) may at first seem contradictory to previous studies linking θ oscillations to social-related processes (Blume et al., 2015). However, as frontal θ activity has been associated with multiple cognitive and affective processes beyond social cognition—cognitive control, working memory, sustained attention (Cavanagh and Frank, 2014), as well as emotional regulation (Ertl et al., 2013)—this finding should be interpreted cautiously. If perspective-taking is viewed as an automatic and effortless cognitive process, one possible explanation could be that higher cognitive perspective-taking during ad viewing may reflect a more fluent processing of the testimonial interaction, thus associated with reduced cognitive control, workload (Chikhi et al., 2022), and mental fatigue (Wascher et al., 2014). However, because cognitive control, workload, and mental fatigue were not directly measured in this study, this interpretation should be regarded as a post-hoc hypothesis rather than evidence of these mechanisms. This interpretation may also be consistent with the *embodied simulation* framework for social cognition, which suggests that understanding others relies on automatic, rapid, internal representations of observed behaviors and emotions (Gallese, 2007). Within this framework, perspective-taking during ad viewing may involve the spontaneous internal simulation of the testimonial's experience, facilitating the processing of the observed interaction.

The observed positive correlations between COG and the clustering coefficient in the α band (*C*_α_) may suggest the involvement of cognitive processes related to information integration during ad viewing. Previous studies have associated α-band connectivity with working memory processes (Dai et al., 2017), and have related working memory to visuo-spatial perspective-taking (Zhang et al., 2022). In addition, α-band clustering coefficient connectivity patterns have already been linked to social cognition mechanisms and to social stimuli processing (Maffei et al., 2023). Consistently, reduced α activity has been observed in clinical populations showing impairments in social cognitive functioning (Yun et al., 2024), or altered emotional processing and regulation (Shim et al., 2018). At the same time, it should be noted that α-band clustering has also been associated with other processes, such as behavioral inhibition and control (Tian et al., 2021). Therefore, while these findings may reflect processes related to social cognition and perspective-taking, their functional interpretation in terms of working-memory processes should be considered with caution and only as a plausible hypothesis based on previous literature, rather than as a direct demonstration of working memory involvement in the task.

Similar considerations may apply to the positive correlations between perceived ad effectiveness (EFF) and the clustering coefficient in the α band (*C*_α_). As previous evidence has suggested that working memory processes can contribute to advertising effectiveness (Gordon et al., 2018), the observed association may reflect cognitive processes involved in the processing and evaluation of the ad. However, as α connectivity has been linked to multiple functions—as previously discussed (e.g., Yun et al., 2024; Shim et al., 2018; Tian et al., 2021)—this interpretation should be treated as tentative. Regarding the positive correlation between EFF and kappa divergence in the α band (*K*_α_), it may reflect differences in network organization during the processing of ads perceived as more effective. Previous studies indeed identified a larger *K*_α_ in contexts associated with reward-related behaviors, such as internet (Wang et al., 2019) and smartphone (Yin et al., 2024) overuse, have been discussed in relation to reward-related mechanisms (Poisson et al., 2021). Nevertheless, our study did not directly assess reward processing, thus this functional interpretation of the kappa divergence finding should be considered as one of multiple possible explanations to be tested in future research.

Still regarding α connectivity measures, EFF was associated with characteristic path length—as observed for the δ band. Although longer path length is typically linked to reduced efficiency and performance (van den Heuvel et al., 2009), it may instead reflect higher working memory and a deeper, more elaborative processing, in line with the interpretation of the previous connectivity results. Higher path length has indeed been found in younger vs. older individuals, with better support for more demanding processes (Vecchio et al., 2017). This interpretation is further supported by the positive correlation between EFF and the average degree, suggesting that the perception of an effective ad is linked to a more connected network. Such metric has been related to emotional awareness (Smith et al., 2018) and task-related processing vs. resting states (Wadhera and Kakkar, 2021). In particular, Wadhera and Kakkar (2021) also links the average degree to social cognition processes, further supporting a connection between perceived ad effectiveness and social cognition. However, the interpretation of these connectivity measures is not univocal. Given that they have been associated with multiple, even contradictory processes, and that working memory, elaborative processing, and emotional awareness were not directly assessed in this study, they should be treated as only one of several possible explanations that require confirmation in future research.

It should also be noted that the observed effects were not consistent across all advertising stimuli. No significant correlations emerged for AD3, and only a limited number of effects were observed for AD1 and AD_2_. These differences may partly reflect variation across stimuli in product type, brand, testimonial characteristics, and interaction type (eating vs. drinking), all of which are known to influence consumers' responses to advertising (Martin et al., 2008; Graham and Wilder, 2020) and may also affect perspective-taking processes. At the same time, the heterogeneity of these stimulus features does not allow the identification of more specific patterns, such as effects linked to a particular product category or testimonial gender. In addition, the analyzed interaction segments were relatively short and varied in duration, which may have influenced the EEG measures (Fraschini et al., 2016; van Vugt et al., 2007). Spectral results also showed limited statistical strength and were restricted to specific electrodes (i.e., F7 and F4), rather than extending to broader scalp regions such as the left and right frontal areas. These findings may reflect the well-known limitations of scalp EEG relative to source-level analyses (Hatlestad-Hall et al., 2023; Song et al., 2015), as well as the limited statistical power of the present study, which may have increased the risk of failing to detect true effects (Button et al., 2013). Overall, the observed effects should therefore be interpreted with caution.

Although exploratory, our findings suggest a possible partial overlap between EEG correlates of cognitive perspective-taking and perceived ad effectiveness, particularly in relation to the clustering coefficient in the α band. It could be interpreted as a preliminary indication that the two constructs may rely on partly related neurophysiological patterns, consistently with frameworks such as the Narrative Transportation Theory, which posits that identification with characters (i.e., through perspective-taking processes) can enhance ad's impact (Green and Brock, 2000). In testimonial-based advertising, perspective-taking with the character may therefore represent one possible process contributing to ad evaluations and ad effectiveness. This result addresses the third research objective and demonstrates how combining EEG spectral features with connectivity measures can enrich our understanding of consumer social cognition processes.

### 4.1 Practical implications

From a more applied point of view, these preliminary findings suggest that EEG-based spectral and connectivity measures may provide complementary information for advertising evaluation and consumer research. In particular, these measures could be useful in advertising pre-testing by complementing traditional self-report evaluations. For example, they could help identify which ad segments, product-interaction scenes, or testimonial characteristics are associated with stronger consumer perspective-taking (as well as other deeper, psychological processes) or perceived effectiveness. This information could support comparisons between different versions of the same advertisement, different narrative structures, or different testimonial profiles, helping practitioners better understand which elements of the ad are more engaging for consumers. However, given the exploratory nature of the present study, these measures should not be considered standalone indicators of ad effectiveness, and their practical use in both academic and everyday market research practice should be validated in larger studies including behavioral or market-relevant outcomes.

### 4.2 Limitations and future research

Despite offering preliminary insights into the EEG correlates of perspective-taking in relation to ad effectiveness, this study calls for cautious interpretation and suggests several directions for future research.

As already discussed, the stimulus set included only four ads that differed in several advertisement-specific characteristics such as content, product category (food vs. beverage), testimonial gender and characteristics, emotional nature, narrative style, context, interaction type, and production style. Although the stimuli were intentionally selected to preserve a naturalistic advertising context, these factors may have independently influenced both EEG and self-report responses (Vecchiato et al., 2011; Russo et al., 2023), making it difficult to attribute the observed correlations specifically to perspective-taking rather than to particular features of the individual ads. Therefore, factors such as product category, testimonial gender, emotional content, and narrative style should also be considered as potential confounding variables in the interpretation of the present findings. The use of relatively short ad clips may have also limited the ecological validity of the findings, as real-world exposures often involve longer and more continuous narrative content. This study did not assess individual differences that may shape responses to advertising stimuli, such as perspective-taking ability (both cognitive and emotional), empathy, brand familiarity, or engagement with the advertised product or brand (Ciorciari et al., 2019). Including such measures could help explain the differences observed in both EEG responses and subjective evaluations. Future studies could therefore employ a larger and more controlled set of stimuli, including longer and more continuous narrative content, and ideally allowing for a systematic manipulation or matching of advertisement-specific characteristics such as brand identity (Graham and Wilder, 2020), narrative style (Simonetti et al., 2024), testimonial gender (Martin et al., 2008), and the degree of similarity between the viewer and the testimonial (e.g., gender or other shared characteristics), to assess their influence on both neural and reported measures of perspective-taking and help reduce the risk that the observed EEG and self-report effects are driven by uncontrolled stimulus characteristics. It would also be interesting to employ this approach to explore emerging topics, such as how AI content or AI-generated testimonials might affect perspective-taking, as well as overall ad perception and effectiveness. Understanding brain responses to AI-generated versus real content (Bilucaglia et al., 2025) could bring interesting implications for future advertising strategies.

From a statistical point of view, several limitations must be acknowledged. Due to the exploratory nature of the study (Schumm et al., 2013; Bender and Lange, 1999), the final sample size was relatively small, particularly considering the large number of EEG-derived variables and self-report measures analyzed. Additionally, the significance level was lowered (i.e., α = 0.10) and the number of tests performed across EEG and self-report measures—although corrected by FDR—was high. All these aspects may have reduced statistical power. Although the performed sensitivity analysis attempted to estimate the minimum detectable effect sizes, its reliability was limited by the complexity of the analyses performed. The marginally significant findings observed in this study, together with the possibility that some of them may not reflect true effects, are consistent with the characteristics of underpowered studies, which are associated with higher false-negative rates and lower positive predictive value (Button et al., 2013). For these reasons, the present results should be interpreted as preliminary and hypothesis-generating rather than confirmatory. In turn, their generalizability is very limited.

Future studies should employ a larger sample size of at least 40 participants, in line with recent guidelines for EEG in advertising research (Van Diepen et al., 2024). Additionally, the significance should be lowered to more conservative levels (e.g., α = 0.05) and the multiple comparisons should be corrected according to less conservative approaches, such as the cluster-based permutations (Maris and Oostenveld, 2007).

A methodological consideration concerns the level of analysis adopted for both spectral and connectivity measures, which in the present study was the scalp rather than the cortex. Although scalp-level analysis is not uncommon, especially in clinical settings (Seo et al., 2024), its results should be interpreted more cautiously than those obtained at the cortical level (Lai et al., 2018). While source localization and cortical-level functional connectivity can mitigate some limitations of sensor-based measures, such as volume conduction and reference choice, they are considered unreliable when based on fewer than 32 and 64 electrodes, respectively (Hatlestad-Hall et al., 2023; Song et al., 2015). A future confirmatory study using HD-EEG recordings (Stoyell et al., 2021) should therefore verify the present findings at the source level. Another methodological concern relates to the estimation of functional connectivity, which was based on relatively short segments (~3*s*) and may, therefore, be less stable. Although short windows (1−−10*s*) can better capture functional connectivity during continuous, dynamic, non-time-locked stimuli such as video advertisements (Coelli et al., 2025), longer epochs (≥6*s*) may provide more reliable estimates (Fraschini et al., 2016) and should, therefore, be considered in a future study.

Finally, the absence of behavioral measures directly assessing processes such as working memory, cognitive load, attention, and reward processing, limits the possibility of validating the functional interpretation of the observed EEG patterns. Future studies should apply the data triangulation approach (Casiraghi et al., 2026) to neurophysiological and behavioral measures of these processes (e.g., eye-tracking metrics or memory and recall tasks), to allow a more reliable interpretation of the observed neural patterns in terms of specific cognitive mechanisms. Furthermore, additional self-report measures could be implemented to capture deeper aspects of participants' engagement with the ad content. These could comprise narrative transportation and perceived presence, which the literature links to the recruitment of perspective-taking processes and to the enhancement of different aspects of ad perception (Escalas, 2004; Bhatnagar and Wan, 2011).

### 4.3 Conclusions

This exploratory study investigated the relationship between brain activity and consumer responses to advertisements, focusing on cognitive and affective perspective-taking processes and perceived ad effectiveness. Combining EEG spectral power, MST-based connectivity metrics, and subjective ratings, it provided preliminary evidence of distinct yet partially overlapping neural patterns underlying these processes. Although these findings should be considered highly preliminary and interpreted with considerable caution, they offer initial insights into how neural responses may relate to perspective-taking and advertising effectiveness. Some of them seem to align with existing literature on cognitive effort, working memory, and reward processing, while others point to less effortful, perhaps automatic, forms of cognitive engagement during ad viewing. However, these constructs were not directly measured and should, therefore, be treated as hypotheses for future confirmatory research. The results finally highlight the potential of neuroscientific methods—and particularly EEG—in exploring the psychological processes behind consumer perception and behavior, and offer a promising approach for future interdisciplinary research on consumer research and social neuroscience.

## Data Availability

The raw data supporting the conclusions of this article will be made available by the authors, without undue reservation.
